# Eosinophil-Mediated Immune Control of Adult Filarial Nematode Infection Can Proceed in the Absence of IL-4 Receptor Signaling

**DOI:** 10.4049/jimmunol.1901244

**Published:** 2020-07-17

**Authors:** Nicolas Pionnier, Hanna Sjoberg, Julio Furlong-Silva, Amy Marriott, Alice Halliday, John Archer, Andrew Steven, Mark J. Taylor, Joseph D. Turner

**Affiliations:** Centre for Drugs and Diagnostics, Department of Tropical Disease Biology, Liverpool School of Tropical Medicine, Liverpool L3 5QA, United Kingdom

## Abstract

Helminth infections are accompanied by eosinophilia in parasitized tissues. Eosinophils are effectors of immunity to tissue helminths. We previously reported that in the context of experimental filarial nematode infection, optimum tissue eosinophil recruitment was coordinated by local macrophage populations following IL-4R–dependent in situ proliferation and alternative activation. However, in the current study, we identify that control of chronic adult filarial worm infection is evident in IL-4Rα–deficient (IL-4Rα^−/−^) mice, whereby the majority of infections do not achieve patency. An associated residual eosinophilia was apparent in infected IL-4Rα^−/−^ mice. By treating IL-4Rα^−/−^ mice serially with anti-CCR3 Ab or introducing a compound deficiency in CCR3 within IL-4Rα^−/−^ mice, residual eosinophilia was ablated, and susceptibility to chronic adult *Brugia malayi* infection was established, promoting a functional role for CCR3-dependent eosinophil influx in immune control in the absence of IL-4/IL-13–dependent immune mechanisms. We investigated additional cytokine signals involved in residual eosinophilia in the absence IL-4Rα signaling and defined that IL-4Rα^−/−^/IL-5^−/−^ double-knockout mice displayed significant eosinophil deficiency compared with IL-4Rα^−/−^ mice and were susceptible to chronic fecund adult filarial infections. Contrastingly, there was no evidence that either IL-4R–dependent or IL-4R–independent/CCR3/IL-5–dependent immunity influenced *B. malayi* microfilarial loads in the blood. Our data demonstrate multiplicity of Th2-cytokine control of eosinophil tissue recruitment during chronic filarial infection and that IL-4R–independent/IL-5– and CCR3-dependent pathways are sufficient to control filarial adult infection via an eosinophil-dependent effector response prior to patency.

## Key Points

- Immunity to chronic filarial worm infection is apparent in IL-4Rα–deficient mice.
- Delayed immunity in IL-4Rα^−/−^ mice is due to suboptimal tissue eosinophilia.
- Eosinophil recruitment in the absence of IL-4R signaling requires CCR3 and IL-5.

## Introduction

Eosinophilia is a hallmark of the immune response against helminth parasite infection (1–5). Eosinophils are recruited to tissue sites of parasitism and have direct targeted effects on nematode worms, such as degranulation (release of granule constituents such as cytotoxic molecules or enzymes) (2, 6, 7), Ab-dependent cytotoxicity capacity (8, 9), or granuloma formation. The latter is a host protective immune mechanism by which layers of innate effector cells concatenate around excreting–secreting pores of the worm to limit worm motility and enhance effects of granulocyte degranulation (5, 10–12). Neutrophil granulocytes release extracellular DNA “traps” when in contact with *Litomosoides sigmodontis*, *Onchocerca volvulus*, or *Dirofilaria immitis* filarial worms or their endosymbionts, *Wolbachia* (13–15). Because eosinophils can also generate extracellular traps (16, 17), it is postulated that eosinophils may also contribute toward this antifilarial effector immune response.

The mechanisms coordinating eosinophil recruitment to the site of infection remain to be resolved. In a previous investigation, we reported that macrophages polarized to an alternatively activated phenotype through IL-4R activation mediated immunity to the human lymphatic filarial pathogen, *Brugia malayi*, by sustaining a vigorous CCR3-dependent eosinophilia at the site of infection (18). We demonstrated that this mechanism substantially influenced numbers of infectious-stage larvae surviving to the immature adult developmental stage.

In the current study, we explored the long-term consequences of IL-4Rα deficiency and degree of eosinophil recruitment on *B. malayi* filarial parasitism using a typically nonpermissive BALB/c mouse model of infection and additional IL-5 and CCR3 compound deficiencies.

## Materials and Methods

### Mice, infections, and treatments

BALB/c male mice were purchased from Charles River Laboratories. BALB/c IL-4R chain α knockout (IL-4Rα^−/−^) and CCR3^−/−^ mice were purchased from The Jackson Laboratory. BALB/c IL-4Rα^−/−^/CCR3^−/−^ double-knockout mice were created by crossing IL-4Rα^−/−^ and CCR3^−/−^ mice. BALB/c IL-4Rα^−/−^/IL-5^−/−^ mice were a gift from Professor A. Hoerauf, University Hospital Bonn. *Meriones unguiculatus* (Mongolian gerbils) were originally purchased from Charles River Laboratories. All knockout mouse lines and gerbils were subsequently bred in-house. All animals were maintained in specific pathogen-free conditions at the University of Liverpool Biological Services Unit. The experimental life cycle of *B. malayi* was maintained by passage between i.p. infections of male gerbils and membrane-feeding of Liverpool strain *Aedes aegypti* mosquitoes, as previously described (19), to provide infectious third-stage larvae or microfilariae (mf) for infections.

Groups of between four and six mice of 6–10 wk of age were used for experimental infections. *B. malayi* third-stage larvae were counted in batches of 50 in RPMI medium and inoculated via i.p. injection (IP) as previously described (19). Certain mouse groups were treated with either 0.1 mg rat IgG IP (control) or 0.5 mg anti-αCCR3 Ab (clone: 6S2-19-4; Bio X Cell) IP to deplete CCR3^+^ eosinophils. All experiments on animals were approved by the ethical committees of Liverpool School of Tropical Medicine and the University of Liverpool and were conducted according to Home Office Legislation, the revised Animals (Scientific Procedures) Act of 1986, and Animal Research: Reporting of In Vivo Experiments guidelines. *B. malayi* mf were purified from the peritoneal washings of infected gerbils as previously described (19). *B. malayi* mf were concentrated by centrifugation and resuspended at a density of 1.25 × 10^6^/ml in RPMI medium before batches of 0.25 × 10^6^ were infused i.v. via the tail vein.

### Parasites and cells collection

Motile *B. malayi* adult stage parasites and peritoneal exudate cells were recovered by peritoneal lavage at necropsy and worms were enumerated by microscopy. At ≥11 wk postinfection, peritoneal mf numbers were determined through peritoneal lavage with 1 ml RPMI and total mf count in a 10-μl suspension.

Presence of mf in the peripheral circulation or in the cardiopulmonary system of infused mice was detected through thick smears and subsequent Giemsa staining as previously described (20). Briefly, 20 μl of blood from the tail vein (as for peripheral blood microfilaremia) or 50 μl from the heart (at necropsy, as for cardiopulmonary microfilaremia) were processed for a thick smear through a scratch method and slides were then stained with 40% Giemsa.

### Flow cytometry

After parasite enumeration and removal, peritoneal exudates were centrifuged (250 G, 5 min, 4°C), and cells were resuspended in 1 ml FACS buffer (PBS, 5% FCS, and 1 mM EDTA). Total cell counts were performed in PBS/0.04% trypan blue (Sigma-Aldrich) using a hemocytometer (KOVA Glasstic Slide). Proportions of most of the different leukocyte populations were determined by flow cytometry using the following rat anti-mouse Abs: anti-F4/80–BV711 (clone BM8; BioLegend), anti–Siglec F PE (clone E50-2440; BD), anti–Ly-6G–BV650 (clone 1A8; BioLegend), anti-B220–PE-Cy5 (clone RA3-6B2; eBioscience), anti-CD3–AF700 (clone 17A2; eBioscience), anti-IgE–FITC (clone 23G3; Invitrogen), and anti-CD49b–APC (clone DX5; eBioscience). For innate lymphoid cell populations, a lineage mixture comprised anti-CD8 (clone 53-6.7; eBioscience), anti-B220 (clone RA3-6B2; eBioscience), anti-F4/80 (clone BM8; eBioscience), anti–Siglec F (clone ES22-10D8; Miltenyi Biotec), anti-CD4 (clone GK1.5; eBioscience), anti–Ly-6G (clone RB6-8C5; eBioscience), and anti-FcƐR1 (clone MAR-1; eBioscience) Abs conjugated to APC were used in combination with anti-ST2–PE (clone RMST2-2; eBioscience), anti-NKp46–AF700 (clone 29A1.4; BD), anti–IL-12Rβ2–AF488 (clone 305719; R&D), and anti–CD127 PerCP-Cy5.5 (clone A7R34; eBioscience). Eosinophil activation markers panel comprised anti-F4/80–BV711 (clone BM8; BioLegend), anti–Siglec F APC (clone REA798; Miltenyi Biotec), anti-CD28–PE-Cy7 (clone 37.51; Thermo Fisher Scientific), anti–MHC class II (MHCII)–AF700 (clone M5/114.15.2; Thermo Fisher Scientific), anti-CD86–BV605 (clone GL-1; BioLegend), anti-CD69–APC eF780 (clone H1.2F3; Thermo Fisher Scientific), and anti-CCR3–PE (clone J073E5; BioLegend) Abs. All panels included a viability staining using the eF450 viability dye (Invitrogen), and all Abs were used at a 1/40 dilution. Representative gating strategies are given in Supplemental Figs. 1, 2. Flow cytometric acquisition was performed on a BD LSR II and data analyzed on FlowJo Software (version 10.0.7).

### Statistical analysis

Statistical analysis was carried out using GraphPad Prism v8. Sample size, normality (Shapiro–Wilk test), and homoscedasticity (Bartlett test) were tested prior to further analysis. Data from two to three separate experiments were pooled when possible. Significant differences between groups were evaluated by either *t* test (two groups) or Mann–Whitney (more than two groups) or Kruskal–Wallis with Dunn post hoc tests (more than two groups). Significance was defined as *p < 0.05, **p < 0.01, ***p < 0.001, and ****p < 0.0001.

## Results

### IL-4Rα–independent immunity is sufficient for protection against chronic adult *B. malayi* infection

**FIGURE 1.**
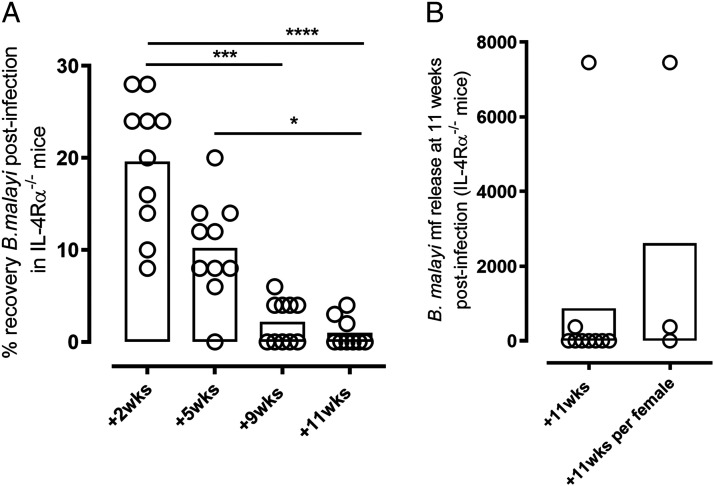
Gradual waning of patent adult worm infections in *B. malayi*–infected IL-4Rα^−/−^ mice. (**A**) Parasite recovery at 2, 5, 9, and 11 wk after *B. malayi* infection in IL-4Rα^−/−^ mice. Kruskal–Wallis one-way ANOVA followed by Dunn multiple comparisons test (*n* = 9), two individual experiments). *p < 0.05, ***p < 0.001, ****p < 0.0001. (**B**) mf release in the peritoneal cavity of IL-4Rα^−/−^–infected mice at readout 11 wk postinfection (*n* = 9), two individual experiments.

Recently, we have defined a mechanism of adaptive immune control against invading *B. malayi* filarial larvae that is mediated by IL-4–IL-4R alternative activation of peritoneal macrophages and resultant CCR3-dependent tissue eosinophilia (18). Key timings of the *B. malayi* life cycle developmental stages in mice are: third–fourth stage larvals molt around days 7–10, fourth stage larvals–adult stage molt around 4–5 wk, and mf release from females at the site of infection (patent phase) from week 11 onwards. We thus examined IL-4Rα^−/−^ mouse susceptibility to immature (prefecund) adult *B. malayi* starting at 5 wk postinfection (Fig. 1A) when the majority of wild-type (WT) mice have cleared infections at a point preceding adult development (11, 18, 21). Because severe-combined lymphopenic strains lacking functional adaptive immunity are fully susceptible to chronic patent *B. malayi* infections (11, 18, 19, 21–24), we therefore scrutinized whether specific deficiency in IL-4Rα signaling was sufficient to also allow progression of development of *B. malayi* adults toward an early fecund time-point of 11 wk postinfection (Fig. 1A). During time course infection experiments, although infection is beginning to be cleared in WT animals at 5 wk postinfection (mean 2% parasite recovery and sterilizing immunity evident in ∼50% of animals) (18), 9/10 IL-4Rα^−/−^ mice contained adult *B. malayi*, and levels of motile parasites were not significantly different to numbers of surviving larvae at 2 wk postinfection (Fig. 1A). At 9 wk postinfection, 5/10 mice had no evidence of infection, and yields of recovered adults were significantly reduced compared with 2-wk larval yields (median yield of 2 versus 22 *B. malayi*; Kruskal–Wallis one-way ANOVA statistic 28.4; *p* < 0.001 Dunn multiple comparisons test). By 11 wk postinfection infections are resolved in WT mice (18). Similarly, the majority of IL-4Rα^−/−^ mice (6/9) were infection negative, and levels of surviving adults were significantly reduced compared with larval burdens at 2 wk or adult loads at 5 wk (median yield of 0 versus 22 *B. malayi* at 2 wk, *p* < 0.0001; or versus 10 *B. malayi* at 5 wk, *p* < 0.05). mf production was only detected in 2/9 mice at 11 wk in 2/3 mice with active adult infection (Fig. 1B), indicating that sterile cure of adult *B. malayi* had occurred in the majority of IL-4Rα^−/−^ mice at a point prior to establishment of fecund infections.

### IL-4Rα–independent immunity to fecund adult *B. malayi* is associated with a residual CCR3-mediated eosinophilia

**FIGURE 2.**
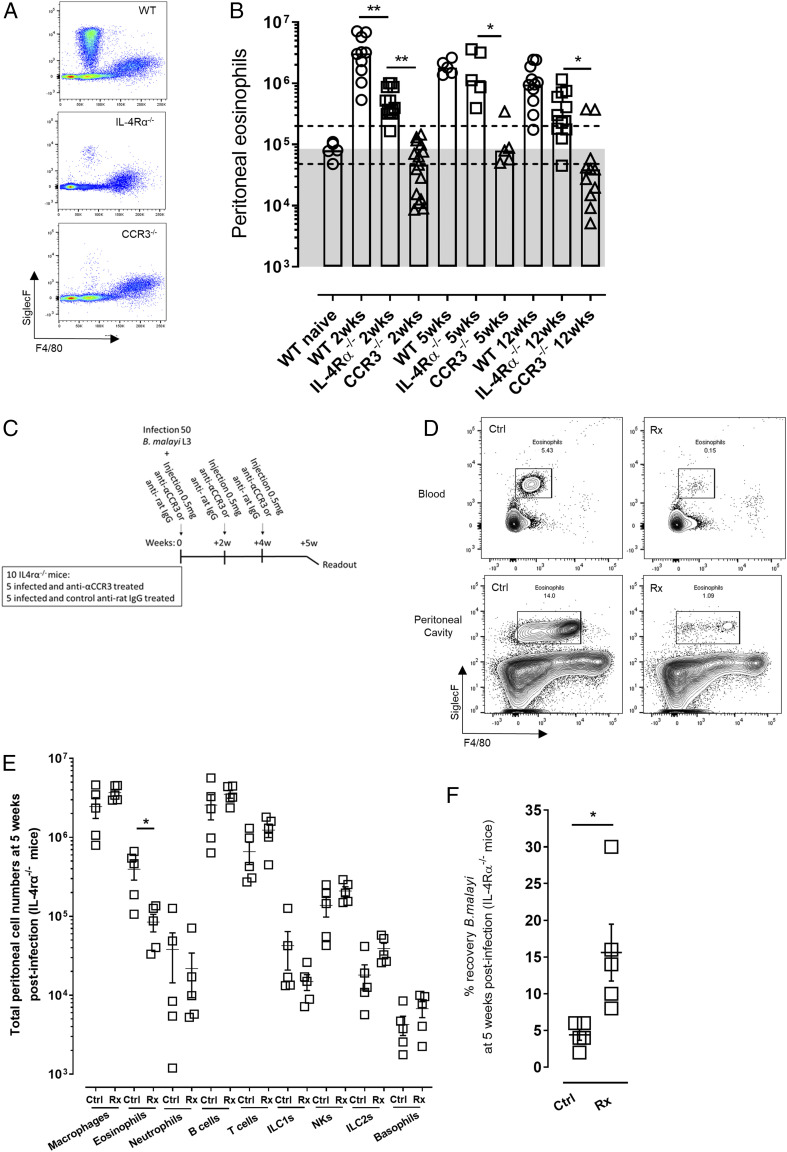
Temporal ablation of residual eosinophilia enhances survival of *B. malayi* adult infection in IL-4Rα^−/−^ mice. (**A**) Representative flow cytometry plots for eosinophils phenotyping based on their Siglec F marker expression (Siglec F^+^ and F4/80^low^) 12 wk postinfection in infection WT, IL-4Rα^−/−^, and CCR3^−/−^ mice. (**B**) Peritoneal eosinophil numbers in either WT, IL-4Rα^−/−^, or CCR3^−/−^ mice at 2, 5, and 12 wk after *B. malayi* infection. Kruskal–Wallis one-way ANOVA with Dunn multiple comparisons tests or Mann–Whitney tests (*n* = 5–15), one to two individual experiments. The area within the two dashed lines represents ±2-fold change of WT naive peritoneal eosinophil levels, and data falling above the gray area indicate increased eosinophil numbers compared with WT naive mice. *p < 0.05, **p < 0.01. (**C**) Schematic representation of the study design for temporal CCR3-dependent eosinophilia ablation using an anti-αCCR3 blocking Ab. (**D**) Representative plots of blood and peritoneal eosinophils successful ablation at 2 wk after anti-αCCR3 blocking Ab injection at 0.5 mg in IL-4Rα^−/−^ mice (*n* = 4). (**E**) Comparative peritoneal cell population numbers at 5 wk postinfection in infected IL-4Rα^−/−^ mice and either treated with anti-αCCR3 blocking Ab (Rx) or its respective isotype control (Ctrl). Kruskal–Wallis one-way ANOVA with Dunn multiple comparisons tests (*n* = 5), single experiment. *p < 0.05. (**F**) Parasitical readout at 5 wk postinfection in IL-4Rα^−/−^ mice injected either with Rx or its Ctrl. *t* Test, (*n* = 5), single experiment. *p < 0.05.

Because immune resistance to filarial infection is dependent on a sustained influx of eosinophils mediated by the CCR3-CCR3 ligand chemokine pathway (1, 4, 11, 18, 21), the levels of Siglec F^+^ eosinophils in the peritoneal infection site were compared between WT, IL-4Rα^−/−^, and CCR3^−/−^ mice (Fig. 2A, 2B). At 2 wk postinfection, corresponding with initial larval *B. malayi* establishment, median levels of eosinophils were elevated >30-fold compared with naive basal levels in the peritonea in BALB/c WT mice (median level: 3.03 × 10^6^ at 2 wk postinfection versus 0.09 × 10^6^ in naive mice; Fig. 2B). Contrastingly, eosinophilia was not apparent in infected CCR3^−/−^ mice (median level: 0.04 × 10^6^). In comparison, IL-4Rα^−/−^ mice exhibited a suboptimal eosinophilia 2 wk postinfection, which despite being >9-fold significantly impaired compared with WT mice, was nonetheless significantly elevated compared with CCR3^−/−^ animals (0.36 × 10^6^ median level, Kruskal–Wallis one-way ANOVA statistic: 41, *p* < 0.01 compared with WT or CCR3^−/−^ mice, Dunn multiple comparisons tests; Fig. 2B). The residual eosinophilia was maintained throughout the time course of infection in IL-4Rα^−/−^ mice and was significantly elevated compared with corresponding CCR3^−/−^-infected animals at 5 wk (*p* = 0.05) or 12 wk postinfection (*p* = 0.05; Dunn multiple comparisons tests; Fig. 2B). To determine whether this residual eosinophil population in IL-4Rα^−/−^ mice was immunophenotypically different to the one of their WT mice counterparts, we examined eosinophil-related CCR3, CD28, MHCII, CD86, CD69, and RELMα markers expressions in WT and IL-4Rα knockout mice at 14 d postinfection (Supplemental Fig. 2). We did not detect any significant differences in eosinophil phenotypes in the absence of IL-4R signaling, suggesting that these eosinophils populations are not immunologically different. To find out whether residual eosinophil recruitment was important in mediating gradual immune-dependent attrition of adult *B. malayi* in the face of IL-4R deficiency, we depleted eosinophils using a rat monoclonal anti-(α)CCR3 Ab treatment known to transiently ablate mature eosinophil populations, including those recruited to sterile sites of filarial infection (18, 25, 26). Groups of mice were administered with αCCR3 or rat isotype control every 2 wk, commencing from the point of infection with *B. malayi* larvae (Fig. 2C). One week following the final dose, at 5 wk postinfection, levels of eosinophils were significantly reduced (by >4-fold) in the peritonea of infected, αCCR3-treated IL-4Rα^−/−^ mice compared with isotype-treated infection controls (from 0.398 × 10^6^ to 0.085 × 10^6^ mean levels, respectively, Kruskal–Wallis one-way ANOVA statistic: 80.61, Dunn multiple comparisons tests, *p* < 0.05; Fig. 2D, 2E). By comprehensive immunophenotyping, all other major populations of leukocytes in the peritonea of infected IL-4Rα^−/−^ mice were found to be unperturbed by the course of Ab treatment (Fig. 2E). The impact of serial αCCR3 treatments and eosinophil ablations was a significant increase in the yield of immature *B. malayi* adults at 5 wk postinfection (mean worm recovery rate of 4.2 and 15.5 for control and treated mice, respectively, Student *t* test, *t*: 2.843, *p* = 0.0217; Fig. 2F).

**FIGURE 3.**
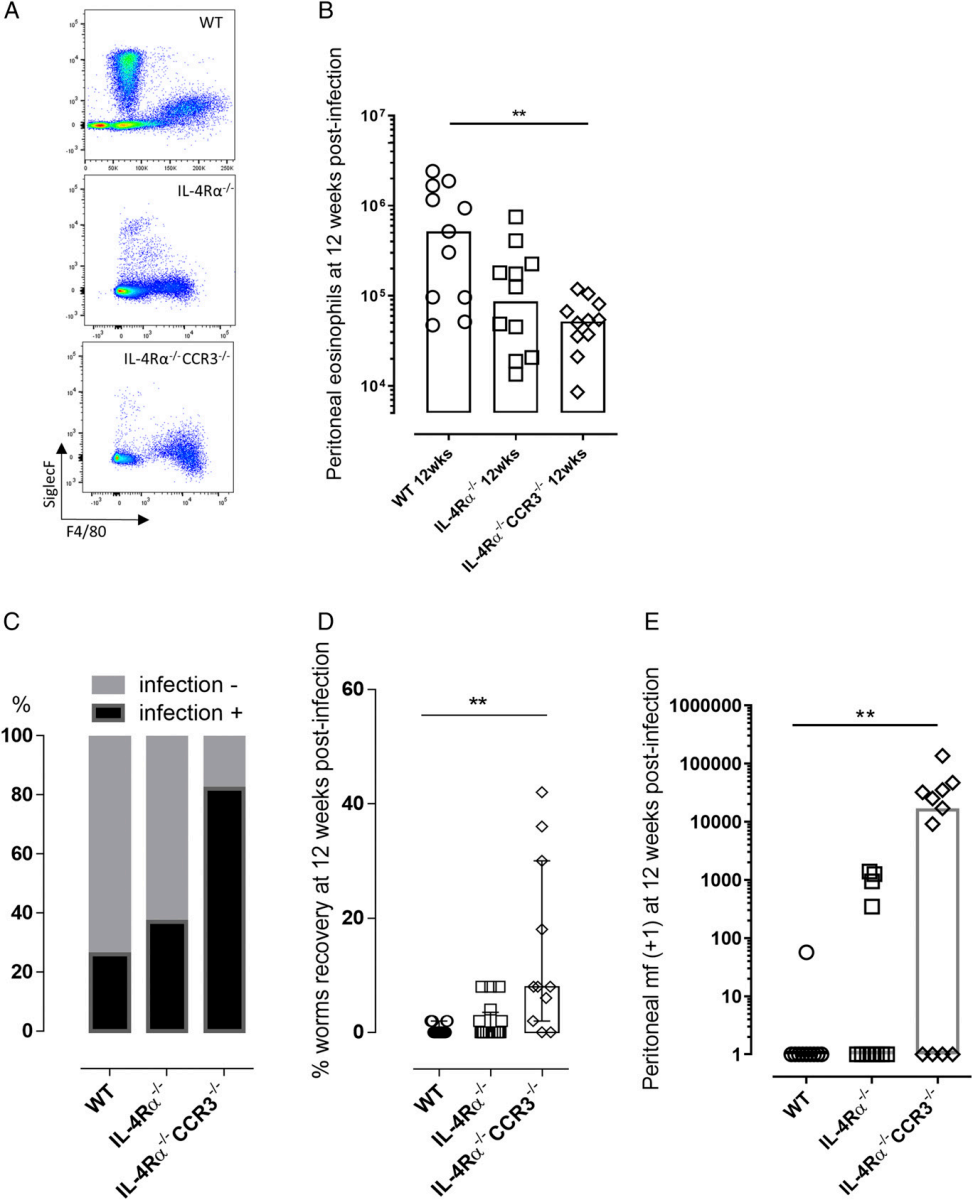
CCR3 signaling is required for residual eosinophilia and resistance to chronic fecund *B. malayi* infection in IL-4R–deficient mice. (**A**) Representative flow cytometry plots for eosinophils and macrophages phenotyping based on their Siglec F and F4/80 respective marker expression (eosinophils being Siglec F^+^ F4/80^low^, whereas macrophages are Siglec F^low^ F4/80^+^) at 12 wk postinfection in infected WT, IL-4Rα^−/−^, and IL-4Rα^−/−^CCR3^−/−^ mice. (**B**) Comparative peritoneal eosinophilia at 12 wk after *B. malayi* infection in WT, IL-4Rα^−/−^, and IL-4Rα^−/−^CCR3^−/−^ mice. Kruskal–Wallis one-way ANOVA followed by Dunn multiple comparisons tests (*n* = 10–11), two individual experiments. **p < 0.01. (**C**) Percentage of mice still infected 12 wk after third-stage infectious larvae inoculations χ^2^ analysis, *p* < 0.0001, *n* = 10–11, two individual experiments. (**D**) Worm recoveries at 12 wk postinfection in infected WT, IL-4Rα^−/−^, and IL-4Rα^−/−^CCR3^−/−^ mice. Kruskal–Wallis one-way ANOVA with Dunn multiple comparisons tests or Mann–Whitney tests (*n* = 10–11), two individual experiments. **p < 0.01. (**E**) mf release in the peritoneal cavity of infected WT, IL-4Rα^−/−^, and IL-4Rα^−/−^CCR3^−/−^ mice at 12 wk postinfection. Kruskal–Wallis one-way ANOVA followed by Dunn multiple comparisons tests (*n* = 10–11), two individual experiments. **p < 0.0.

In a complementary approach, and because it was not possible to sustainably administer Ab treatments for longer than a 5-wk period, we created an IL-4Rα^−/−^CCR3^−/−^ double-knockout mouse on the BALB/c background. This enabled us to further scrutinize whether IL-4Rα^−/−^–independent sterilizing immunity required CCR3-mediated recruitment of peritoneal eosinophils. At 12 wk postinfection (Fig. 3), eosinophilia was significantly impinged in IL-4Rα^−/−^CCR3^−/−^ double-knockout mice compared with WT infections, whereas as IL-4Rα^−/−^ mice exhibited an intermediate eosinophilia that was not significantly inferior to eosinophil levels in WT mice (median eosinophil levels of 0.52 × 10^6^, 0.087 × 10^6^, and 0.05 × 10^6^ for WT, IL-4Rα^−/−^, and IL-4Rα^−/−^CCR3^−/−^ mice, respectively; Kruskal–Wallis one-way ANOVA statistic 10.3, *p* < 0.01; Fig. 3A, 3B). In WT and IL-4Rα^−/−^ groups, the majority of mice had no evidence of active adult infection (9/11, WT; and 6/10, IL-4Rα^−/−^), and remaining infected animals contained a single live adult *B. malayi*. In comparison, a significantly higher proportion (9/11) of IL-4Rα^−/−^CCR3^−/−^ double-knockout mice yielded adult worm infections (χ^2^ statistic: 70.61, *p* < 0.0001; Fig. 3C, 3D). The resultant *B. malayi* burden in IL-4Rα^−/−^CCR3^−/−^ mice was significantly increased compared with WT or IL-4Rα^−/−^ groups (median percentage recovery of 8, range 0–42, Kruskal–Wallis one-way ANOVA statistic: 14.2, *p* < 0.05 versus WT mice, *p* < 0.01 versus IL-4Rα^−/−^ mice, Dunn multiple comparisons tests; Fig. 3D). Further, *B. malayi* fecund infections were evident in (7/11) IL-4Rα^−/−^CCR3^−/−^ mice versus 4/11 IL-4Rα^−/−^ mice and 1/10 WT mice (Fig. 3E). Levels of mf production in the peritonea of IL-4Rα^−/−^CCR3^−/−^ mice were significantly elevated compared with WT mice (median 17 × 10^3^ peritoneal mf versus 0, Kruskal–Wallis one-way ANOVA statistic 9.5, *p* < 0.01, Dunn multiple comparisons test; Fig. 3E). Therefore, our infection model indicated CCR3-dependent processes and recruitment of residual eosinophilia were sufficient to exert immune control of fecund infections in the absence of IL-4R signaling.

### IL-5 is critical in the regulation of residual eosinophilia and immunity against adult *B. malayi* in the absence of IL-4R signaling

**FIGURE 4.**
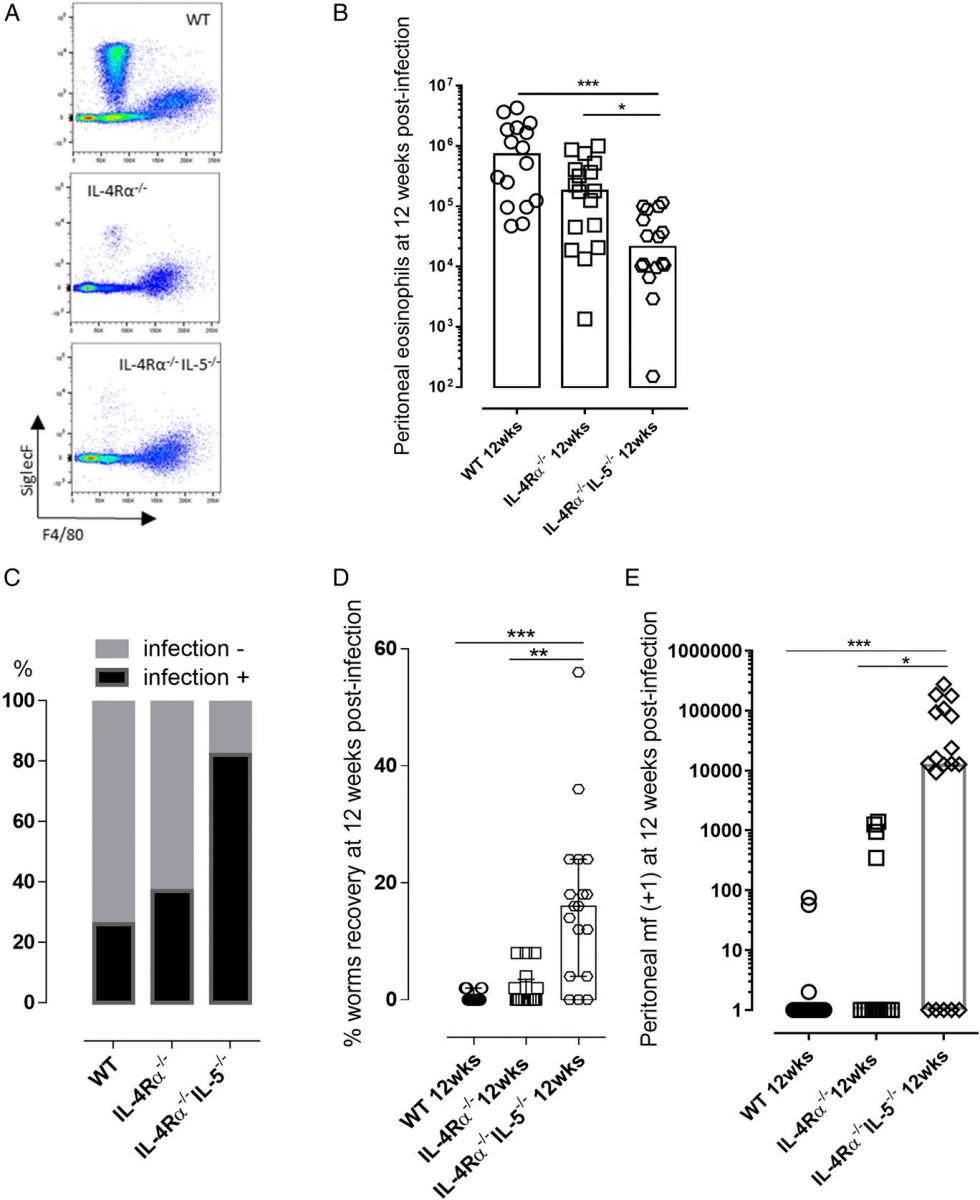
IL-5 is required for residual eosinophilia and resistance to chronic fecund *B. malayi* infection in IL-4R–deficient mice. (**A**) Representative flow cytometry plots for eosinophil and macrophage phenotyping based on their Siglec F and F4/80 respective marker expression 12 wk postinfection in infected WT, IL-4Rα^−/−^, and IL-4Rα^−/−^IL-5^−/−^ mice. (**B**) Comparative peritoneal eosinophil at 12 wk after *B. malayi* infection in WT, IL-4Rα^−/−^, and IL-4Rα^−/−^IL-5^−/−^ mice. Kruskal–Wallis one-way ANOVA followed by Dunn multiple comparisons tests (*n* = 16–19), three individual experiments. *p < 0.05, ***p < 0.001. (**C**) Percentage of mice still infected 12 wk after third-stage infectious larvae inoculations χ^2^ analysis, *p* < 0.0001 (*n* = 16–19), three individual experiments. (**D**) Worms recoveries at 12 wk postinfection in infected WT, IL-4Rα^−/−^, and IL-4Rα^−/−^IL-5^−/−^ mice. Kruskal–Wallis one-way ANOVA with Dunn multiple comparisons tests or Mann–Whitney tests (*n* = 16–19), three individual experiments. **p < 0.01, ***p < 0.001. (**E**) mf release in the peritoneal cavity of infected WT, IL-4Rα^−/−^, and IL-4Rα^−/−^IL-5^−/−^ mice at 12 wk postinfection. Kruskal–Wallis one-way ANOVA followed by Dunn multiple comparisons tests (*n* = 16–19), three individual experiments. *p < 0.05, ***p < 0.001.

IL-5 is important in the immune control of filarial infection (27–30). We and others have shown that *Brugia* experimental infections stimulate high levels of IL-5 systemic responses (18, 21, 31). IL-5 is a promoter, survival, and chemotactic factor for eosinophils (1, 27, 32). To explore whether IL-5 was a necessary component of IL-4Rα^−/−^–independent immunity to adult filarial infection, we compared susceptibility at the fecund adult stage 12 wk postinfection in WT, IL-4Rα^−/−^, and IL-4Rα^−/−^IL-5^−/−^ mice (Fig. 4, Supplemental Fig. 2). We determined eosinophilia was profoundly impaired in IL-4Rα^−/−^IL-5^−/−^ mice compared with either WT or the residual eosinophil level in IL-4Rα^−/−^ mice (median eosinophilia 0.02 × 10^6^ versus 0.7 × 10^6^, WT or 0.18 × 10^6^, IL-4Rα^−/−^, Kruskal–Wallis one-way ANOVA statistic: 21.5, *p* < 0.001 or *p* < 0.05, Dunn multiple comparisons tests; Fig. 4A, 4B). The majority of IL-4Rα^−/−^IL-5^−/−^ mice contained active adult worm infections and frequency of infection was significantly higher compared with WT or IL-4Rα^−/−^ mice (15/18 mice, versus 5/19 WT and 6/16 IL-4Rα^−/−^ mice, χ^2^ statistic: 70.61, *p* < 0.0001; Fig. 4C). Adult *B. malayi* worm burdens were significantly higher in IL-4Rα^−/−^IL-5^−/−^ mice compared with either WT or IL-4Rα^−/−^ animals (median percentage recovery 16 versus 0 in both WT or IL-4Rα^−/−^ mice, Kruskal–Wallis one-way ANOVA statistic: 22.4, *p* < 0.001 or *p* < 0.01, Dunn multiple comparisons tests; Fig. 4D). Fecundity, measured by presence of motile peritoneal mf, was discernable in 13/18 IL-4Rα^−/−^IL-5^−/−^ mice, 3/19 WT mice, and 7/16 IL-4Rα^−/−^ mice. The yields of mf produced were significantly higher in IL-4Rα^−/−^IL-5^−/−^ mice than either WT or IL-4Rα^−/−^ animals (median mf count 13 × 10^4^ versus 0 for both WT and IL-4Rα^−/−^ groups, Kruskal–Wallis one-way ANOVA statistic: 19.3, *p* < 0.001 versus WT or *p* < 0.05 versus IL-4Rα^−/−^, Dunn multiple comparisons tests; Fig. 4E).

### Deficiency in IL-4R signaling alone or combined with deficiency in CCR3 or IL-5 does not influence microfilarial infection

**FIGURE 5.**
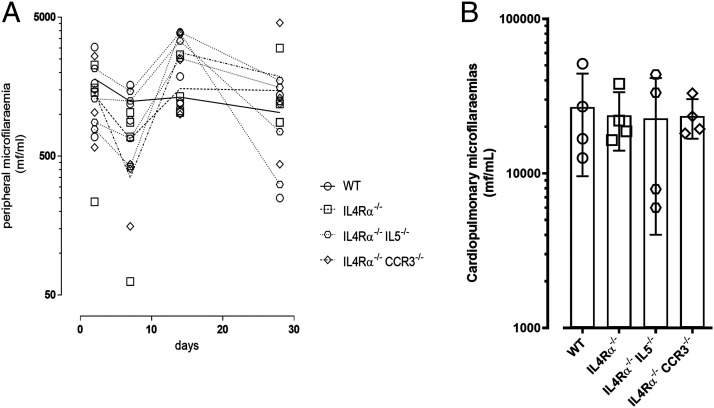
IL-4R deficiency alone or combined with IL-5 or CCR3 deficiencies do not significantly alter *B. malayi* microfilaremias. (**A**) Comparative peripheral blood microfilaremia over a 28-d time course in WT, IL-4Rα^−/−^, IL-4Rα^−/−^IL-5^−/−^, and IL-4Rα^−/−^CCR3^−/−^ mice infused with 250000 *B. malayi* mf at day 0 (*n* = 4), single experiment. (**B**) Comparative cardiopulmonary blood microfilaremia at 28 d after *B. malayi* mf infusion in WT, IL-4Rα^−/−^, IL-4Rα^−/−^IL-5^−/−^, and IL-4Rα^−/−^CCR3^−/−^ mice (*n* = 4), single experiment.

We defined that IL-4R–independent, CCR3, and IL-5 eosinophil recruitment processes limited patent adult filarial infections. In the minority of IL-4Rα^−/−^ mice featuring active fecund infection, yields of mf produced were lower, either reflecting an indirect effect of reduced adult burdens or, alternatively, that IL-4Rα–independent processes were also directly affecting mf survival. Because distinct immune responses are triggered against different life cycle stages of filarial parasites (1, 10, 29, 33, 34), we scrutinized whether either IL-4R–dependent or IL-4R–independent/CCR3- or IL-5–dependent immunity was also operating at the level of stage-specific mf infections. We infused mice with 0.25 × 10^6^ motile *B. malayi* mf and analyzed circulating parasitemias over a time course of 28 d. Microfilaremias were stable in WT mice over the time course (Fig. 5A). No significant increases were apparent in circulating parasitemias at any time-point within IL-4Rα^−/−^, IL-4Rα^−/−^CCR3^−/−^, or IL-4Rα^−/−^IL-5^−/−^ mice compared with WT controls. Because the majority of surviving infused *B. malayi* mf sequester in the cardiopulmonary circulation (19, 20), the levels of mf in cardiac blood at termination were also evaluated (Fig. 5B). Similar microfilaremias were recorded between WT and IL-4Rα^−/−^, IL-4Rα^−/−^CCR3^−/−^, or IL-4Rα^−/−^IL-5^−/−^ mice, indicating that these immunodeficiencies did not have any impact on mf parasitism in the blood during the first 4 wk of a primary infection.

## Discussion

A high degree of compensatory pathways and functional redundancy are apparent in Th2 immunity. In the current study, we reinforce the primary role of eosinophilia in driving immune control of adult filarial nematode infection, yet demonstrate that eosinophil-mediated immune resistance can proceed in the absence of IL-4R signaling. We demonstrate that a residual eosinophilia occurs in *B. malayi*–infected IL-4Rα^−/−^ mice, which we successfully targeted via serial treatment with anti-αCCR3 depleting Ab. This ablating Ab treatment did not impact on any other local effector immune cell type examined and led to an increased susceptibility to *B. malayi* infection.

IL-4 and IL-13 are drivers of key hallmarks associated with type-2 inflammation (18, 35, 36). Both *il-4* and *il-13* gene activation can be mediated through the IL-4Rα (37). Both cytokines are, for instance, critical for helminth infection-associated intestinal mucus production (38, 39), *Trichinella spiralis*–dependent host inducible nitric oxide synthase expression (40, 41), positive regulation of the Foxp3^+^ T cell populations with the tissue-dwelling parasites *Schistosoma mansoni* and *Nippostrongylus brasiliensis* (42), and optimal *Heligmosomoides polygyrus* expulsion (43). In the context of filariasis, IL-4 and IL-13 have been described as critical cytokines required for the expansion of tissue-resident macrophages that optimally maintain eosinophilic influx and effector responses at the site of infection (18, 26). Nonetheless, IL-4/IL-13 helminth-associated immune mechanisms are parasite species specific with, for example, goblet cell hyperplasia impairment observed with *N. brasiliensis* experimental infections in IL-4Rα^−/−^ mice, but not with *Syphacia obvelata* and *S. mansoni* infection settings (39). Our present study also indicates other host-derived mechanisms or cytokines can partially compensate the absence of IL-4R signaling to exert Th2 immunity (36). It has been shown that several cytokines such as TNF-α, IL-1, and TGF-β can contribute to CCL11 production in a context of IL-4Rα deficiency (44). We and others have previously shown that macrophage CCR3 ligand expression (CCL8, CCL11, and CCL24) is upregulated during filarial infection and that macrophages are essential for maintaining eosinophil recruitment into the peritoneal cavity (18, 45). During type 2 inflammation, in the absence of IL-4R, other macrophage ligand–receptor-activating signals have been implicated in macrophage proliferation or alternative activation, such as IL-10, IL-33, and Ig (45–47). Thus, it is possible that IL-4R–independent macrophage activation during chronic filarial infection supports suboptimal eosinophil recruitments sufficient for protection against adult *B. malayi* infection.

Eosinophilia at tissue sites of inflammation can be mediated by a number of distinct molecules or pathways, including ILs such as IL-2, IL-3, and IL-5; growth factors, such as (G)M-CSF; chemokines such as CCL5, 11, 24, and 26; and leukotrienes, such as leukotriene B4 (48, 49). Our data indicate that both CCR3-dependent ligands and IL-5 are necessary to induce eosinophilia to filarial infection in the absence of IL-4R signaling. This may reflect that IL-5 signaling is fundamental to support CCR3 chemotaxis to the peritoneal site of filarial infection. Certainly, increased expression of CCR3 is demonstrable in IL-5–activated eosinophils and augments eotaxin (CCL-11)–mediated eosinophil chemotaxis in vitro (50). Alternatively, IL-5 may be working as an independent chemotactic factor by which both IL-5R ligation and CCR-3 ligation are nonredundant signals combining to mediate peritoneal tissue eosinophilia in response to filarial parasitism. A recent study with *L. sigmodontis* infections comparing IL-4Rα^−/−^ with IL-4Rα^−/−^IL-5^−/−^ combined deficiency also recorded residual eosinophilia in IL-4Rα–deficient mice, which was ablated when compound IL-5 deficiency was introduced and was significantly related to increased susceptibility to fecund adult infections (19). IL-5 residual immune responses also are important in the control of juvenile adult development of the related human filaria *Loa loa* during IL-4Rα deficiency (51).

Beyond activation and recruitment to the site of infection, both CCR3 ligands and/or IL-5 may augment tissue eosinophil survival. Following *N. brasiliensis* or *T. spiralis* infection, the rate of eosinophil production in the bone marrow is unaltered, whereas pronounced eosinophilia is recorded in several tissues, suggesting that recruited eosinophil survival is increased (52, 53). Murine eosinophils migrating to the peritoneum could recirculate to other organs, thus indicating the peritoneum as a reservoir for eosinophils (52).

Eosinophils have prior been defined as an essential effector cell component of the antifilarial immune response against various filarial parasite life cycle stages in vitro and in vivo (54), including an active involvement in direct mf killing (2, 55), in granulocytic content release at the site of infection (2, 4, 56), mf-targeted Ab-dependent cytotoxicity mechanisms (55, 57), and granuloma clustering around infective and adult stages (10, 12, 58). Our present study illustrates that whereas a muted eosinophil response is evident in the absence of IL-4/IL-13 signaling, low eosinophilia is still an integral component of immune attrition to juvenile adults in IL-4Rα mice. We further characterized eosinophils from IL-4Rα^−/−^ mice and compared them to their WT mice–derived counterparts. We analyzed the expression profiles of the well-defined and inflammation or parasitic infection-driven phenotypic markers, such as the systemic highly upregulated CD86 and CD69 markers (59–61), the blood highly upregulated CD28 marker (59, 60); the lung, blood, and bone marrow upregulated MHCII and RELMα markers (59, 62, 63); and the peritoneum, bone marrow and blood downregulated CCR3 marker (64, 65). Interestingly, expression profiles were very similar, suggesting that these eosinophils populations are not immunologically different. Contrastingly, we did not observe any alterations in circulating *B. malayi* microfilaremias in either IL-4Rα^−/−^ or IL-4Rα^−/−^CCR3^−/−^ or IL-4Rα^−/−^IL-5^−/−^ mice compared with WT controls for a period of up to 4 wk following infusion. This may indicate that these eosinophil recruitment and survival responses are not important in the control of *B. malayi* filarial microfilaremias in the circulation or the spleen. This may appear contradictory with prior work, demonstrating that control of *B. malayi* microfilaremias require CCL-11, eosinophil production, and eosinophil specific granule products (2, 66, 67). However, prior work has shown that deficiency in the CCR3 ligand, CCL-11, renders mice more susceptible to persistent microfilaremias after 49 d (66). It is therefore possible that longer-term primary infections might resolve the relative roles of IL-4Rα, IL-5, and CCR3 in eosinophil-mediated control of circulating mf.

The major cellular sources of CCR3 ligands and IL-5 supporting IL-4Rα–independent, eosinophil-mediated immunity to *B. malayi* was not examined in this study. In the context of helminth infection, IL-5 is known to be produced not only by CD4^+^ T cells but also type 2 innate lymphoid cells (68, 69). In our system, ILC population levels at the site of parasitism were similar between IL-4R–sufficient and –deficient animals at 12 wk postinfection (Supplemental Fig. 3). If IL-4R–independent immune mechanisms occur in the natural human host, targeting of a non–IL-4/IL-13–dependent eosinophilic immune pathway may be of potential benefit in a vaccination approach to derisk the generation of severe anaphylactic, IgE-mediated side effects.

Beyond basic immunobiology, the identification of eosinophilic control of patent adult *B. malayi* infection and its successful manipulation by compound IL-4Rα^−/−^CCR3^−/−^ or IL-4Rα^−/−^IL-5^−/−^ deficiencies offers new selective knockout research models for translational filariasis research. One advantage of a refined selective knockout system, over lymphopenic models currently in use (20, 21), is the maintenance of more realistic serological components (immunoglobulins, immune-complexes, and complement) that may otherwise cause artefacts in the discovery of novel filarial biomarkers.

In summary, our data confirm the pivotal role of eosinophils in the control of the filarial infection and reinforces the importance of CCR3-dependent eosinophilia in mediating immunity to the human filarial pathogen, *B. malayi* (18). Although IL-4R–dependent responses are necessary for rapid eosinophil-mediated primary immunity to establishing filarial larvae, we reveal that a layer of redundancy exists so that IL-4/13–independent and IL-5– and CCR3-dependent processes can compensate and more gradually exert immune killing to prefecund adult infections by mediation of a modified eosinophil recruitment.

## Supplementary Material

Data Supplement
