# Supplementary material for: Eosinophil-Mediated Immune Control of Adult Filarial Nematode Infection Can Proceed in the Absence of IL-4 Receptor Signaling
Source: J Immunol. 2020 Jul 17;205(3):731–40. doi: 10.4049/jimmunol.1901244 (PMC7372315; doi:10.4049/jimmunol.1901244)
Supplement: Data Supplement [file JI_1901244.zip › JI_1901244_Supplemental_Figures_1.pdf]

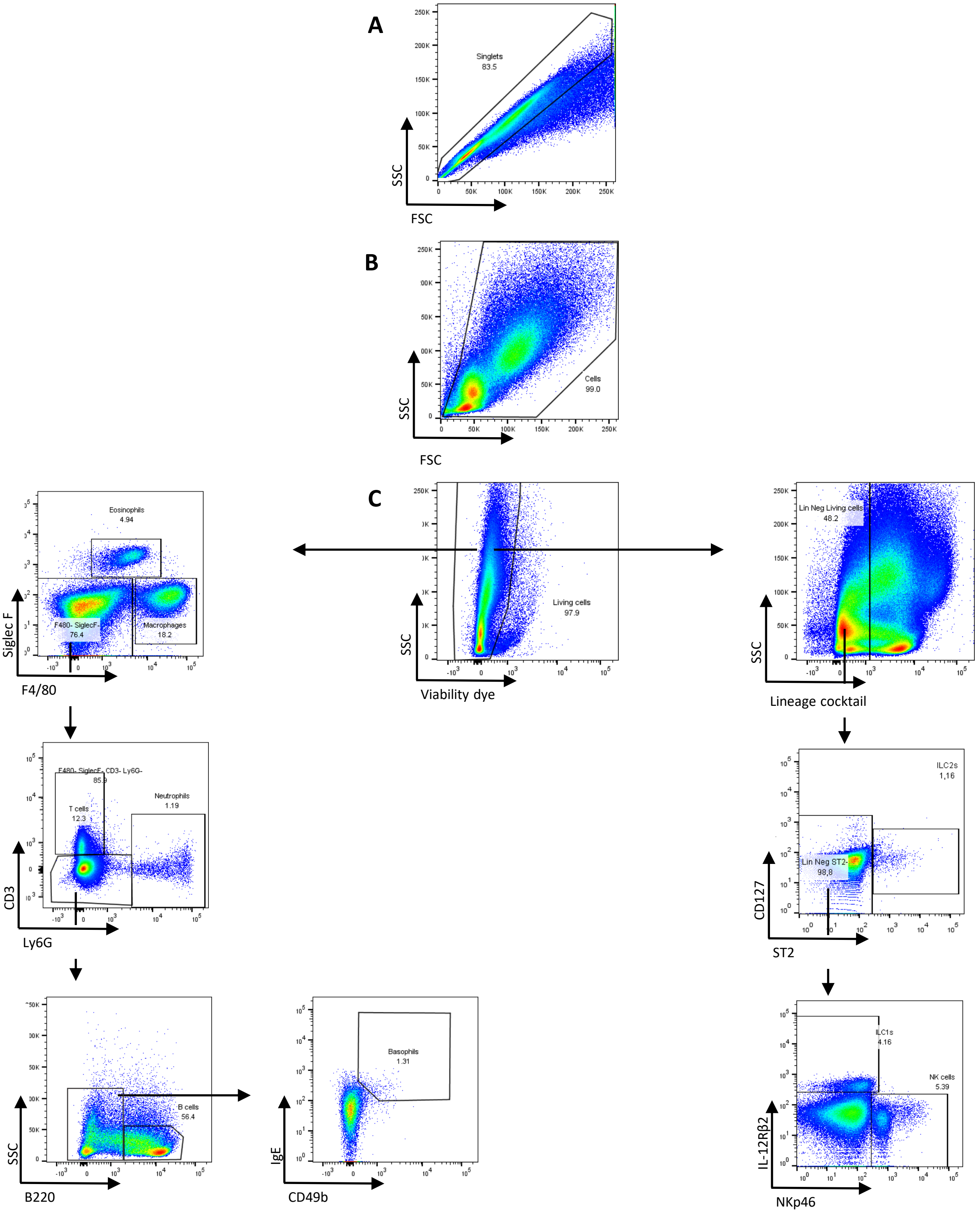

Figure S1: Gating strategy for the phenotyping of the peritoneal cells. Doublets (A), debris (B) and dead cells (C) were excluded and pan markers antibodies were utilised for the different cell populations. Eosinophils were gated as SiglecF^{+} F4/80^{low}; macrophages as SiglecF^{-}F4/80^{high}; neutrophils as Ly6G^{+}SiglecF^{-}F4/80^{-}; T cells as CD3^{+}Ly6G^{-}SiglecF^{-}F4/80^{-}; B cells as B220^{+}CD3^{-}Ly6G^{-}SiglecF^{-}F4/80^{-} and basophils as B220^{-}CD3^{-}Ly6G^{-}SiglecF^{-}F4/80^{-}IgE^{+}CD45^{+}. Innate lymphoid cells were pre-gated on lineage negative cell populations (CD8^{-}, B220^{-}, F4/80^{-}, SiglecF^{-}, CD4^{-}, Ly6G^{-}, FcγR1^{-}) and identified as CD127^{low}ST2^{+} for ILC2s; ST2^{-}IL-12Rβ2^{+}NKp46^{-} for ILC1s and ST2^{-}IL-12Rβ2^{-}NKp46^{+} for NK cells.

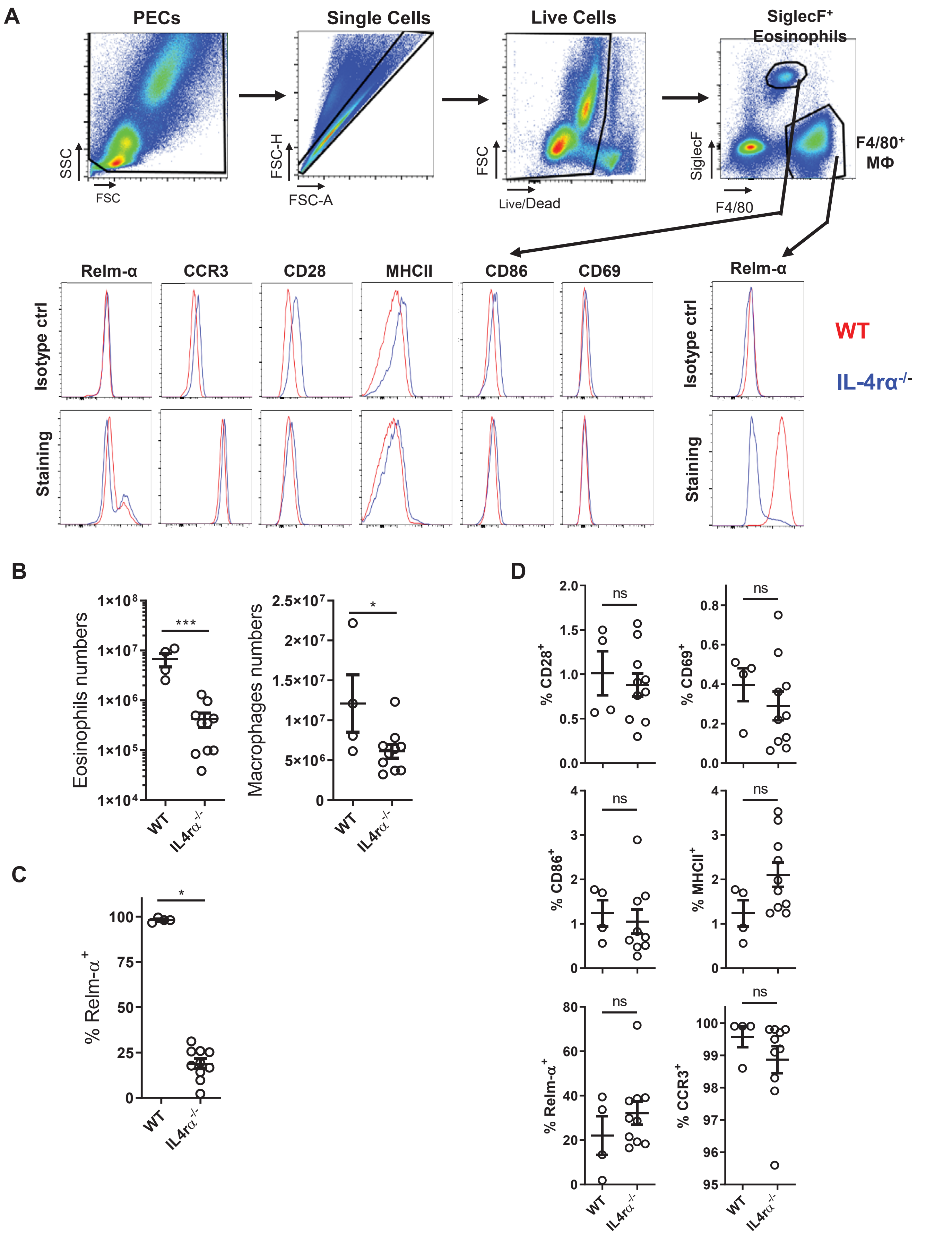

Figure S2: IL-4Rα signalling is required for eosinophil recruitment to filarial infection site but has no significant impact on eosinophil phenotype.
(A) Gating strategy for the immunophenotyping of peritoneal macrophages and eosinophils in WT (red) or IL-4Rα$^{-/-}$ infected mice. (B) Total peritoneal eosinophil and macrophage numbers in WT and IL-4Rα$^{-/-}$ infected mice at 14dpi. (C) Relm-α macrophage expression displayed as percentage of total macrophage population. (D) CD28, CD69, CD86, MHCII, Relmα and CCR3 expression in eosinophil populations coming from either WT or IL-4Rα$^{-/-}$ infected mice at 14dpi. n=4-10, data is from a single experiment with statistical significance indicated as: ***=P<0.001, *=P<0.05, ns= not significant derived from a two tailed independent student T-test.

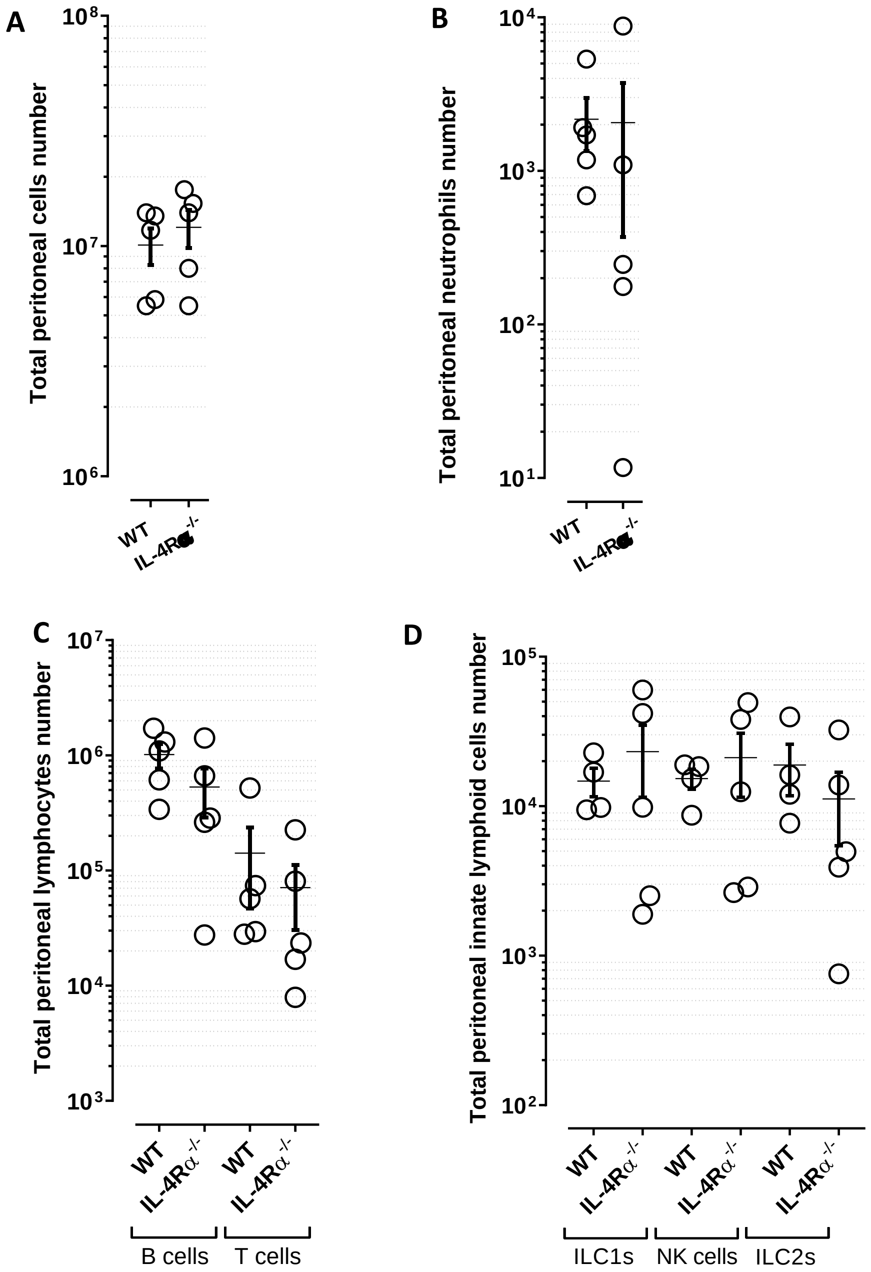

Figure S3: Chronic neutrophil, B cell, T cell and innate lymphoid cell recruitment to the site of infection are not effected by IL-4R deficiency following filarial infection. (A) Comparative total peritoneal cell numbers in BALB/c WT and IL-4rα$^{-/-}$ mice at 12 weeks post *Brugia malayi* infection and their associated composition in neutrophils **(B)**, lymphocytes **(C)** and ILC populations **(D)**. Data were analysed using T tests, n=5, 1 experiment.
